# Selective Covalent Conjugation of Phosphorothioate DNA Oligonucleotides with Streptavidin

**DOI:** 10.3390/molecules16086916

**Published:** 2011-08-15

**Authors:** Kersten S. Rabe, Christof M. Niemeyer

**Affiliations:** TU Dortmund, Fakultät Chemie, Biologisch-Chemische Mikrostrukturtechnik, Otto-Hahn Strasse 6, 44227 Dortmund, Germany

**Keywords:** nanobiotechnology, Protein-DNA conjugate, phosphorothioate, nuclease-stable

## Abstract

Protein-DNA conjugates have found numerous applications in the field of diagnostics and nanobiotechnology, however, their intrinsic susceptibility to DNA degradation by nucleases represents a major obstacle for many applications. We here report the selective covalent conjugation of the protein streptavidin (STV) with phosphorothioate oligonucleotides (psDNA) containing a terminal alkylthiolgroup as the chemically addressable linking unit, using a heterobifunctional NHS-/maleimide crosslinker. The psDNA-STV conjugates were synthesized in about 10% isolated yields. We demonstrate that the terminal alkylthiol group selectively reacts with the maleimide while the backbone sulfur atoms are not engaged in chemical conjugation. The novel psDNA-STV conjugates retain their binding capabilities for both biotinylated macromolecules and the complementary nucleic acid. Moreover, the psDNA-STV conjugate retained its binding capacity for complementary oligomers even after a nuclease digestion step, which effectively degrades deoxyribonucleotide oligomers and thus the binding capability of regular DNA-STV conjugates. The psDNA-STV therefore hold particular promise for applications e.g. in proteome research and novel biosensing devices, where interfering endogenous nucleic acids need to be removed from analytes by nuclease digestion.

## 1. Introduction

Almost 30 years ago, the enormous specificity of Watson-Crick base-pairing of synthetic DNA molecules had been recognized by Nadrian Seeman as a powerful means to rationally construct two- and three-dimensional assemblies from this biopolymer [1]. This groundbreaking research has meanwhile led to the establishment of a vivid and fascinating field of research, often referred to as “DNA nanotechnology” [2], which nowadays can be considered as a mainstream direction in the area of nanobiotechnology [3]. One important topic of DNA nanotechnology and nanobiotechnology concerns the synthesis of semisynthetic conjugates of DNA oligomers and proteins [4]. Such conjugates are highly versatile building blocks because they allow one to combine the unique properties of DNA with the almost unlimited variety of proteins, the latter of which were tailored by billions of years of natural evolution to perform highly specific functions, such as catalytic turnover, energy conversion, or translocation of other components. Consequently, a broad range of applications of semisynthetic DNA-protein conjugates have meanwhile been demonstrated, ranging from nanofabrication of functional devices [4] to clinical diagnostics [5] and the generation of protein microarrays [6]. Covalent DNA conjugates of the biotin-binding protein streptavidin (STV) are particularly noteworthy in the respect, because they combine the high-affinity binding of STV for four molecules of D-biotin with an additional binding site for the complementary nucleic acid. Since their initial description [7], these conjugates have therefore been used as versatile adaptors to readily label biotinylated proteins with an oligonucleotide moiety to realize numerous *in vitro* applications in sensing and biomedical diagnostics [4,6]. However, the intrinsic susceptibility of the tethered DNA oligomers to degradation by nucleases, has so far limited the employment of DNA-STV conjugates for *in vivo* experiments, for instance, in the so-called BioPlex technology, in which cell-penetrating ligands are assembled by means of DNA hybridization to tether combinatorial mixtures of ligands to a plasmid to facilitate cell uptake and gene delivery [8,9]. 

**Figure 1:**
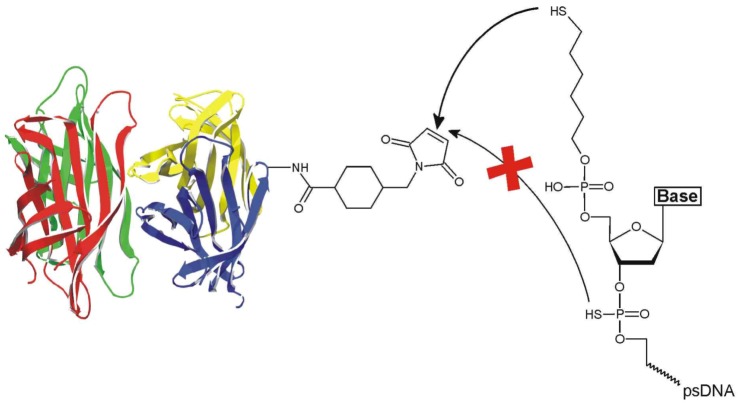
Reaction of sSMCC-activated streptavidin with 5’-C6-alkylthiol-phosphorothioate DNA. Note that the terminal alkylthiol group selectively reacts with the maleimide while the backbone sulfur atoms do not contribute to conjugation.

To overcome the limited stability in complex media, several DNA analogues have been synthesized, which display an increased nuclease stability, such as phosphorothioate DNA (psDNA) [10], PNA, LNA, or morpholino DNA [11]. While peptide conjugates of nucleic acids are accessible by a variety of solid phase-based synthetic approaches [12], to the best of our knowledge, no conjugates of psDNA comprising whole proteins have been described yet. We here report on the synthesis and characterization of well-defined, covalent conjugates of phosphorothioate DNA oligomers and streptavidin (psDNA-STV) (Figure 1). 

Notably, although previous work suggested “considerable thiol character” of the backbone sulfur atoms of the psDNA oligomers [5,13,14], we here demonstrate that psDNA oligomers do not react with the maleimide-based crosslinker sSMCC [4-(N-maleimidomethyl)cyclohexane-1-carboxylic-3-sulfo-*n*-hydroxysuccinimide ester] in the absence of a terminal alkylthiol group, thus giving rise to selective coupling. We demonstrate that the novel psDNA-STV conjugates retain their binding capabilities for both biotinylated macromolecules and the complementary nucleic acid. Owing to the stability of the tethered phosphorothioate oligonucleotide against nucleases, the psDNA-STV conjugates retained their full binding capacity for complementary oligomers even after a nuclease digestion step. In contrast, the nuclease digestion efficiently decreased the binding capacity of regular DNA-STV conjugates. We thus anticipate that the psDNA-STV conjugates will be particularly suited for applications within living cells, such as gene delivery [8], or in proteome research, where interfering endogenous nucleic acids have to be removed from analyte samples by rigorous nuclease digestion [15,16]. Moreover, the robust psDNA-STV conjugates should find applications in the development of novel biosensing devices.

## 2. Results and Discussion

To synthesize the target psDNA-STV conjugates, we adopted the procedure, previously developed for the analogous regular DNA-STV conjugates [7]. In brief, native STV is initially modified with maleimide groups, using an excess of the heterobispecific crosslinker sSMCC. In parallel, any disulfide contaminants within the 5’-alkylthiol-modified phosphothioate DNA, either psF5 (**1**) or psF10 (**2**) oligomers (for sequences, see Table 1) were reduced to enable efficient coupling of the maleimide activated STV. While we had previously optimized this disulfide reduction step with dithiothreitol (DDT) [17], we observed that the psDNA revealed higher coupling efficiency when tris(2-carboxyethyl)phosphine (TCEP) was used as reducing agent. Since TCEP does not interfere with the thiol-maleimide reaction, this change in protocol brought also with it the advantage that no time-consuming separations of the reducing agents are necessary. We first investigated whether the backbone phosphates were also reactive towards sSMCC. To this end, we reacted the psF5 with an excess of sSMCC and butylamine. After purification the reaction products were analyzed by MALDI-TOF. It is shown in Figure S1 that products revealed an increase in mass of 310 *m/z*. Since the observed *m/z* value is in good agreement with the expected mass increase of 293, and the difference of +17 mass units might reflect the presence of an NH_4_^+^-adduct, we concluded that the phosphorothioates exclusively react with the sSMCC at their 5’-alkythiol group. This hypothesis was further confirmed by additional controls with alkylamino-modified psF5, which revealed no reactivity with SMCC-activated STV (see Figure S2). 

**Figure 2:**
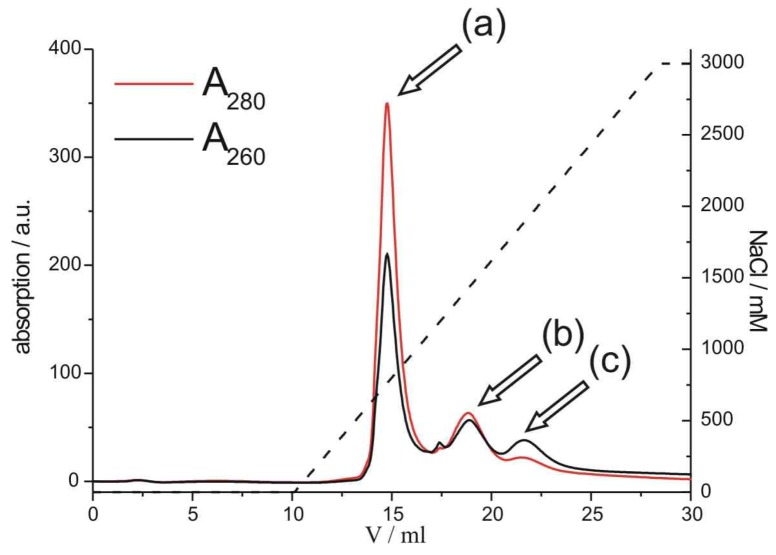
Anion-exchange FPLC chromatogram of the purification of psF5-STV. Streptavidin without psDNA (a) is separated from the psDNA-STV conjugate (b) and free psDNA (c). On the right axis the amount of NaCl in the mobile phase is depicted. Note that the relative absorbances, measured at 280 nm (red line) and 260 nm (black line) are indicative for either the STV or the psDNA-STV conjugate.

In order to covalently couple psF5 or psF10 with STV, the psDNA- and STV-containing solutions were mixed and incubated for 90 minutes. The crude reaction mixture was purified via FPLC on a MonoQ anion exchange column, to separate the psDNA-STV conjugate from remaining unreacted streptavidin (Figure 2). 

Using a linear 0-3 M NaCl gradient, the desired psDNA-STV conjugate [peak (b) in Figure 2] could be separated from unreacted STV [peak (a) in Figure 2] and free DNA [peak (c) in Figure 2]. Similar chromatograms were obtained for the reaction of psF10 with STV (Figure S3). Since the ratio of A260/A280 absorbance is indicative for the coupling stoichiometry, absorbance measurements at 260 and 280 nm and comparison with a calibration curve (Figure S4) indicated that the product in peak (b) represented the psDNA-STV conjugate with a mainly 1:1 stoichiometric ratio of psDNA and STV. The conjugate containing fractions were pooled and concentrated and the isolated yields were determined to be approximately 10%, calculated from the initial amount psDNA. These yields are in the similar range as observed for regular DNA-STV conjugates obtained from deoxyribonucleotides [7,9,17,18] Moreover, a survey of the literature clearly indicates that isolated yields of 10–20% are common average for the synthesis of DNA-protein conjugates, thereby opening the door to a vast variety of practical applications in biosensing and nanosciences [4]. 

**Figure 3:**
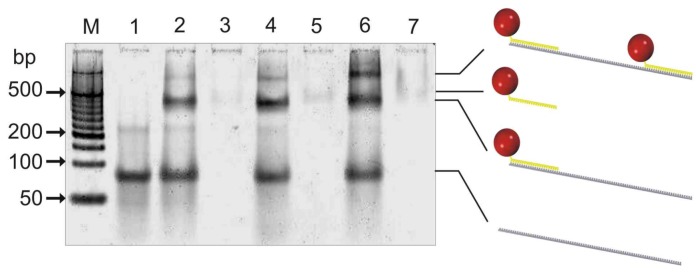
Non-denaturating 8% polyacrylamide gel electrophoresis (ndPAGE) with subsequent SybrGold staining of samples containing psDNA-STV conjugates (5 pmol) and a complementary 103-mer oligonucleotide (10 pmol). Lane M: 50bp MassRuler from Fermentas, lane 1: 103mer alone, lane 2: 103mer and psF5-STV, lane 3: psF5-STV alone, lane 4: 103mer and psF10-STV, lane 5: psF10-STV alone, lane 6: 103mer, psF5-STV, psF10-STV, lane 7: psF5-STV and psF10-STV. The faint band co-migrating with 200 bp marker in lane 1 indicates the presence of low amounts of homodimers of the 103mer oligonucleotide. These homodimers can also bind the STV conjugates leading to the faint bands in lane 2 and 4 which have lower mobility than the 1:1 assembly of conjugate and 103mer. Note that both psDNA-STV do bind to the 103-mer simultaneously (lane 6).

The purity and hybridization capabilities of the resulting psDNA streptavidin conjugates, containing either the F5 or F10 psDNA, were initially analyzed by gel-shift experiments using a non-denaturating polyacrylamid gel (Figure 3). 

**Figure 4:**
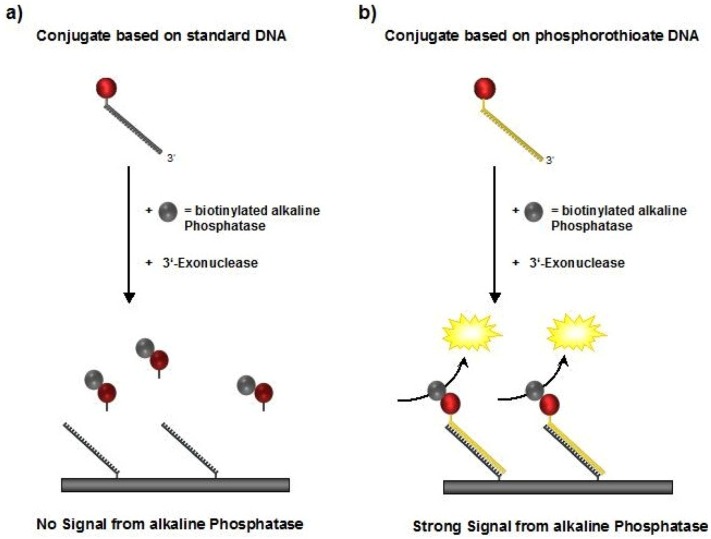
Schematic illustration of the DDI experiments. Biotinylated alkaline phosphatase (bAP) is bound to the STV conjugates in a first step and detected after the DNA-directed immobilization. In case (a) the standard DNA will be digested and the complex is not detectable, whereas the phosphorothioate DNA used in case (b) withstands nuclease treatment, thus resulting in detectable bAP activity.

To this end, psF5-STV or/and psF10-STV were either applied to the gel alone or else mixed with two molar equivalents of a 103-mer oligonucleotide containing sequence stretches, which are complementary to the F5 and F10 psDNA linked to STV. The gel indicated that psDNA-STV conjugates alone (lanes 3, 5, 7) are only weakly stained by SybrGold, a phenomenon also observed for regular ssDNA-STV conjugates [19]. However, strongly stained bands were clearly observable in samples containing the 103-mer oligonucleotide (lanes 1, 2, 4, 6), where bands with a slightly higher electrophoretic mobility as the faint bands of the pure psDNA-STV conjugates suggested that hybridization of the 103-mer strand with the psDNA-STV conjugates readily occurred. When both psF5- and psF10-STV conjugate were present, one additional strong band with decreased mobility was visible (lane 6), indicating the formation of the trimolecular complex, comprising the two conjugates and the 103-mer carrier strand. Although two molar equivalents of the 103-mer carrier were present, the trimolecular complex formed with approximately 40% yield, according to greyscale analysis. This suggests that the hybridization of the psDNA-STV conjugates is not significantly affected by steric hindrance due to binding of both conjugates on the same 103-mer carrier. Concluding, the electrophoresis results therefore confirmed that (i) the psDNA-STV conjugates were synthesized as pure compounds and (ii) that they were capable of specific hybridization with complementary nucleic acids. To further investigate the hybridization properties of the psDNA-STV conjugates, we used DNA-directed immobilization (DDI) in microplates containing complementary capture oligonucleotides, similar as depicted in Figure 4, however, without any nuclease digestion step.

**Figure 5:**
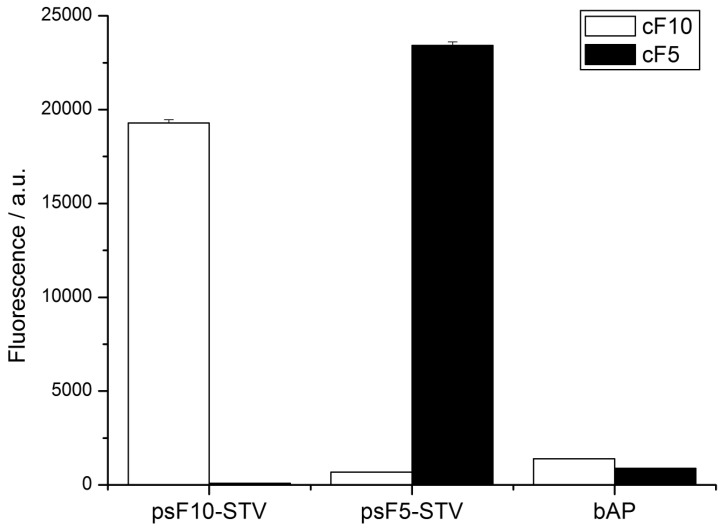
Phosphatase activity detected by AttoPhos assay in a multiwellplate DDI assay. Complementary capture oligonucleotides cF5 or cF10 were immobilized in the wells (black and white bars, respectively), and the indicated samples, containing either psF5- or psF10-STV-bAP conjugates or bAP alone, were allowed to hybridize. Activity of immobilized phosphatase was detected using the AP substrate AttoPhos. Note that signals only occur in wells containing complementary capture oligomers.

To this end, 5’-biotinylated oligomers cF5 and cF10, which are complementary to the psF5- and psF10-STV conjugate, respectively, were immobilized in wells of a streptavidin-coated microplate. The psF5- and psF10-STV conjugates were coupled in homogeneous solution with one molar equivalent of biotinylated alkaline phosphatase (bAP), and the resulting psDNA-STV-AP conjugates were allowed to bind to the microplate-immobilized capture oligonucleotides. Subsequent to hybridization and removal of excess materials, hybridization efficiency was monitored by AP activity, using the fluorogenic substrate AttoPhos. As shown in Figure 5, high AP activity was detected exclusively in wells which contained the complementary capture strand, and no significant cross-hybridization was observed. Also, non-specific adsorption was negligible in controls where only biotinylated alkaline phosphatase was present in the wells. These results demonstrate that the psDNA-STV conjugates possess the similar specificity in DNA recognition as the analogous conjugates, comprising regular DNA oligonucleotides.

**Figure 6:**
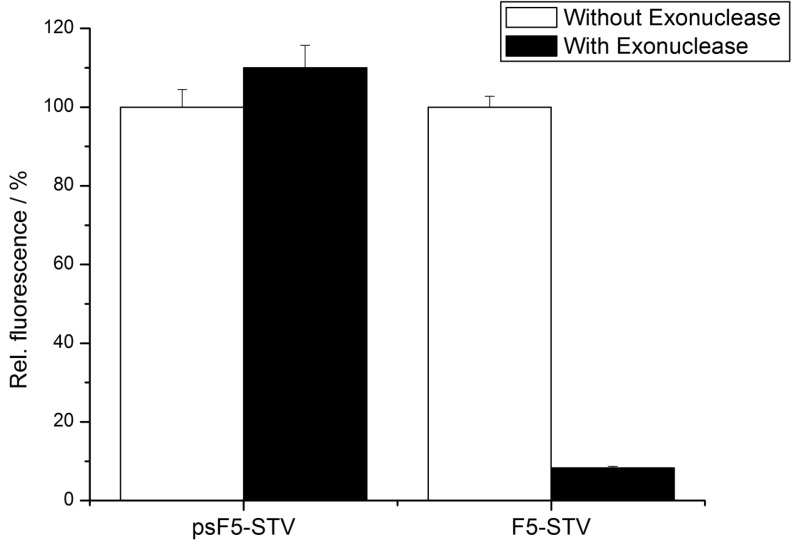
Phosphatase activity detected in the DDI assay depicted in Figure 4 after nuclease digestion by exonuclease I from *E. coli*. Capture oligonucleotides cF5 were immobilized, and the wells were incubated with samples containing either psF5- or F5-STV-bAP conjugates, which were treated (black bars) or not treated (white bars) with exonuclease I digestion. Note that the psF5-STV-bAP retained its full binding capability while the F5-STV-bAP was efficiently degraded by the nuclease.

To experimentally demonstrate the increased stability of the psDNA-STV conjugates against nucleases, we used exonuclease I from *E. coli*. This nuclease is known to efficiently digest single-stranded DNA from the 3’- to the 5’-end. Since linkage of the DNA (psF5 or F5) to the STV occurred via the 5’-thiolgroup, the 3’-end of the oligomers should be susceptible to degradation by Exonuclease I (Figure 4). In contrast, the microplate-bound capture oligomers were immobilized via their 3’-biotinylated ends, which should prevent them from being digested by this nuclease. To investigate nuclease stability, the psF5-STV conjugate as well as a F5-STV conjugate, comprised of standard DNA-bases, were coupled in homogeneous solution with one molar equivalent of biotinylated alkaline phosphatase (bAP). Subsequently half of the sample was treated with exonuclease I to digest the DNA present in the STV conjugate, the other half was left unchanged. The resulting AP-DNA-STV samples were hybridized to microplate-immobilized cF5-capture-oligonucleotides. After removal of unbound materials, the hybridization efficiency was monitored using the fluorogenic substrate Attophos as described before. The resulting data is presented in Figure 6. The phosphatase activity detected for the F5-STV conjugate after digestion with exonuclease I was decreased by more than 90% whereas the activity in case of psF5-STV conjugate was virtually unchanged. This clearly shows that the psDNA conjugates exhibit the expected nuclease stability.

## 3. Experimental

### 3.1. Chemicals and Materials

KH_2_PO_4_, K_2_HPO_4_, TRIS, HCl, NaCl, borate and EDTA were from VWR. The crosslinker sSMCC and tris(2-carboxyethyl)phosphine (TCEP) were purchased from Pierce. DMF and milk powder were supplied by Sigma. β-Mercaptoethanol and Tween 20 were from Roth. Acrylamide solution was purchased at Merck, DNA ladder and loading dye at Fermentas. DTT was from Applichem, Biotin from Fluka and SybrGold from Invitrogen. Exonuclease I was supplied by NEB and biotinylated alkaline phosphatase by Abcam. 5’-C6-alkylthiol-oligonucleotides, 5’-C6-alkylamino-phosphorothioate and 5’-C6-alkylthiol-phosphorothioate olgonucleotides were synthesized by TIB MOLBIOL, the 3’-biotinylated as well as the unmodified oligonucleotides by Sigma. The AttoPhos detection kit was from Roche, the NAP5 and NAP10-columns from GE Healthcare and the Vivaspin concentrators from Satorius.

### 3.2. DNA Sequences

**Table 1:** Oligonucleotide sequences and their corresponding abbreviation in the text.

Number	Name	Sequence
**(1)**	psF5	5'-cggtcataaagcgataag -3', whole backbone is phosphorothioate
**(2)**	psF10	5'-gaatacaaaggctacacg -3', whole backbone is phosphorothioate
**(3)**	103mer	5'-tgtacgtcacaactacttatcgctttatgaccggacccgtgtagcctttgtattcgtccctt
		cacgattgccactttccacgtacttccttaaacgacgcagg-3'
**(4)**	cF5	5'-cttatcgctttatgaccggacc -3'
**(5)**	cF10	5'-cgtgtagcctttgtattcgtcc -3'
**(6)**	F5	5'-ggtccggtcataaagcgataag-3'
**(7)**	cF9	5'-cttcacgattgccactttccac -3'

### 3.3. Synthesis of Covalent Phosphorothioate DNA-sSMCC Conjugates

In an initial test reaction, phosphorothioate psF5-oligonucleotide (1) (20 µL) containing a 5’-C6-alkylthiol group (100 µM in 1 mM TRIS-HCl, 0.1 mM EDTA pH 8.0) was mixed with sSMCC (2 mg dissolved in 60 µL DMF) and incubated for 90 minutes at room temperature in the dark. Subsequently the oligonucleotide-bound sSMCC was reacted with 500 molar excess of butylamine. The reaction product was separated from remaining unreacted sSMCC and butylamine by gel filtration using NAP5 columns. The resulting solution was concentrated to dryness by vacuum concentration, resuspended in H_2_O (20 µL) and 1 µL of the solution was analyzed by MALDI-TOF on a Voyager-DETM Pro Biospectrometry Workstation (Perseptive Biosystems). The spectra are shown in the Supplementary Materials (Figure S1). 

### 3.4. Synthesis, Purification and Characterization of Covalent Phosphorothioate DNA-Streptavidin (psDNA-STV) Conjugates

Phosphorothioate oligonucleotide [100 µL, either psF5 (1) or psF10 (2)] containing a 5’-C6-alkylthiol group (100 µM in 1 mM TRIS-HCl, 0.1 mM EDTA pH 8.0) were activated by the addition of tris(2-carboxyethyl)phosphine (TCEP, 10 µL in 10 mM in 1 mM TRIS-HCl, 0.1 mM EDTA pH 8.0) for 3 hours at room temperature. Streptavidin (200 µL, 100 µM in 50 mM KP_i_, 150 mM NaCl pH 7.4) was mixed with sSMCC (2 mg dissolved in 60 µL DMF) and incubated for 90 minutes at room temperature in the dark. Streptavidin was separated from remaining unreacted sSMCC by two gel filtration steps using NAP5 and NAP10 columns. The eluate was immediately combined with the oligonucleotide and incubated for 90 minutes in the dark. The reaction was then stopped by the addition of 1 M β-mercaptoethanol and the mixture was purified by anion exchange chromatography on an automated FPLC system (Äkta Purifier, GE Healthcare), using a MonoQ 5/5 column equilibrated with 20 mM TRIS/HCl pH 7.5, and a gradient of NaCl ranging from 0 to 3 M (Figure 2). The conjugate containing fractions were pooled and concentrated using Vivaspin concentrators with a molecular weight cut-off of 10.000 Da. Non-denaturing gel-electrophoresis on a 8% polyacrylamide gel (ndPAGE) was used to analyze the purity of the conjugates and their hybridization with complementary DNA oligomers (Figure 3). Electrophoresis buffer was 89 mM Tris, 89 mM Borate, 2 mM EDTA, pH 8.3. To analyze the hybridization properties of the psDNA-STV, 5 pmol of each conjugate was premixed with 10 pmol of a 103mer DNA oligonucleotide (3) containing an 18 base-pair stretch complementary to the phosphorothioate DNA in a total volume of 10 µL in water. The mixture was incubated for 5 minutes at room temperature, mixed with loading dye (2 µL) and applied on the gel. Electrophoresis was carried out for 20 minutes at 20 V/cm and the gel was subsequently stained with SybrGold.

### 3.5. Analysis of the Conjugate Integrity by DNA-Directed Immobilization (DDI)

DDI was carried out as described before, using streptavidin-coated microtiterplates (17). In brief, the plates were washed with TETBS buffer (20 mM Tris, 5 mM EDTA, 150 NaCl, 0.05% (v/v) Tween20), a 240 nM solution of the 5’-biotinylated capture DNA oligonucleotides [cF5 (**4**) and cF10 (**5**), 50 µL] were immobilized, and excess biotin-binding sites were blocked with TETBS containing 0.8 mM D-biotin and 0.45% (w/v) milk powder. Biotinylated alkaline phosphatase (AP, 10 pmol) was mixed with either 10 pmol psF5-STV (**1**), 10 pmol psF10-STV (**2**) or PBS-buffer only in a total volume of 20 µL and incubated for 10 minutes at RT. The blocking buffer was removed from the multiwellplate and after further washing the samples were allowed to hybridize with the surface-bound DNA for 90 minutes. Unbound sample was removed by washing with TETBS twice. AttoPhos detection solution was prepared as described in the suppliers manual and the immobilized complex was then detected through the conversion of the phosphatase substrate AttoPhos by the alkaline phosphatase yielding fluorescent signals, which were recorded using a Synergy multiwellplate reader (BIO-TEK).

### 3.6. Nuclease Digestion Assay

For each sample 10 pmol of either the psDNA-STV [psF5-STV (**2**)] or the analogous regular conjugate [F5-STV, (**6**)], containing a deoxyribonucleic acid moiety, were mixed with biotinylated alkaline phosphatase (AP) in a 1:1 ratio and incubated for 10 minutes at room temperature in 20 µL reaction volume. Subsequently each sample was split into two aliquots, one of which was then subjected to a nuclease digestion. To this end, 10 µL of the sample were mixed with 2.5 µL 10× exonuclease I buffer and 1 µL exonuclease I (20Units/µL) in a total volume of 25 µL and incubated for 30 minutes at 37 °C. The other 10 µL aliquot of the sample were treated in the same way, but omitting the exonuclease I. Subsequently, the samples were diluted with TETBS containing 0.8 mM D-Biotin and 0.45% (w/v) milk powder to a total volume of 100 µL, thus leading to final concentrations of the Oligonucleotide-STV-AP conjugates of 100 nM. Fifty µL of the resulting solution were then allowed to bind to microplate immobilized capture oligomers [either cF5 (**4**) or cF9 (**7**)] prepared as described above. The hybridization event was analyzed by addition of AttoPhos substrate prepared and carried out as described above.

## 4. Conclusions

In this work, we demonstrate that psDNA-STV conjugates can be readily prepared by maleimide-based coupling chemistry. As shown by ndPAGE and DDI, the resulting conjugates display undisturbed hybridization properties. In contrast to standard oligonucleotide DNA-STV conjugates, which are rapidly degraded by DNA nuclease, the psDNA-STV conjugates remained nearly untouched upon treatment with exonuclease I. We anticipate that the synthetic approach described here should be equally applicable to other proteins, thereby enabling access to a new class of sturdy bioconjugates for intracellular or *in vivo* applications in cell biology, bioanalytics or nanobioscience. 

## Supplementary Materials

Supplementary materials can be accessed at http://www.mdpi.com/1420-3049/16/8/6916/s1.
