# Supplementary material for: Selective Covalent Conjugation of Phosphorothioate DNA Oligonucleotides with Streptavidin
Source: Molecules. 2011 Aug 15;16(8):6916–26. doi: 10.3390/molecules16086916 (PMC6264524; doi:10.3390/molecules16086916)
Supplement: Supplementary file 1 [file molecules-16-06916-s001.pdf]

Supporting Information

Selective covalent conjugation of phosphorothioate DNA oligonucleotides with Streptavidin

Kersten S. Rabe and Christof M. Niemeyer

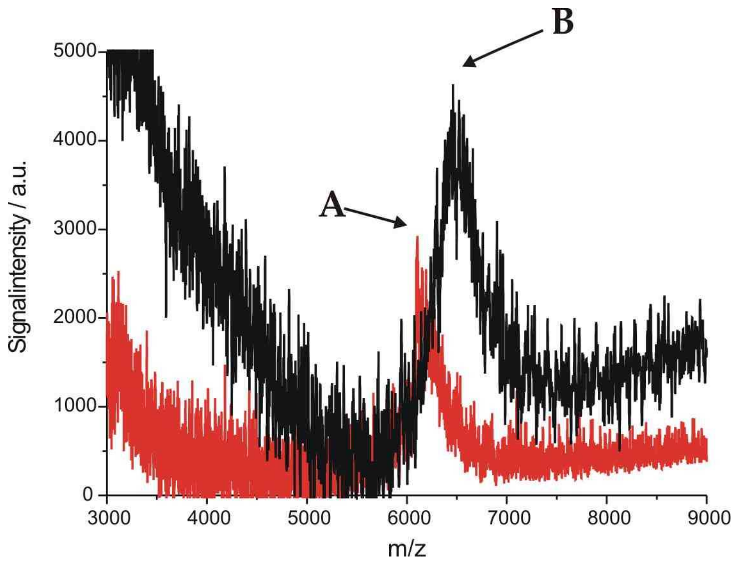

Figure S1: MALDI-TOF analysis of unreacted psF5 (red line) and psF5 (black line) reacted with sSMCC.

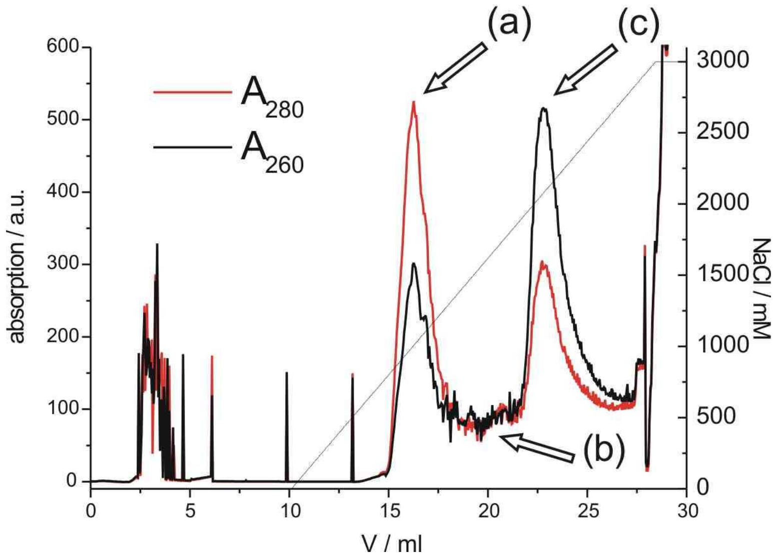

Figure S2: FPLC diagramm of the control reaction of 5'-amino-modified psF1 with maleimide-activated STV. On the right axis the amount of NaCl in the mobile phase is depicted. Streptavidin without psDNA (a) is separated from free psDNA (c). Note that no peak corresponding to the psDNA-STV conjugate (position b, see Figures 2 and S3) could be detected.

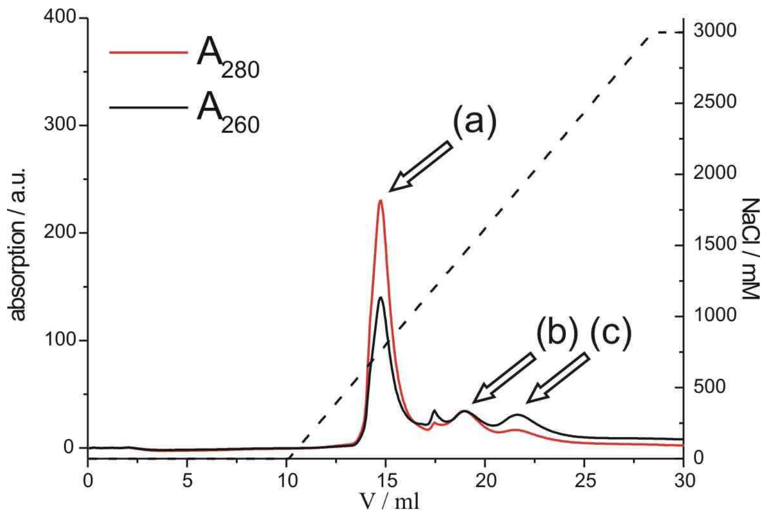

Figure S3: Anion-exchange FPLC chromatogramm of the purification of psF5-STV. On the right axis the amount of NaCl in the mobile phase is depicted. Streptavidin without psDNA (a) is separated from the psDNA-STV conjugate (b) and free psDNA (c).

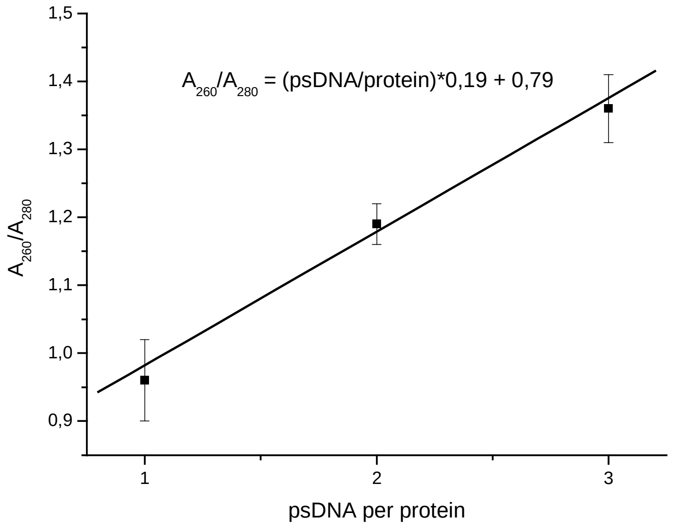

Figure S4: Calibration curve of the ratio of A_{260}/A_{280} absorbance obtained for different mixtures of 3 µM unreacted psF5 and streptavidin.
